# Social risk factors for speech, scholastic and coordination disorders: a nationwide register-based study

**DOI:** 10.1186/s12889-018-5650-z

**Published:** 2018-06-15

**Authors:** Bianca Arrhenius, David Gyllenberg, Roshan Chudal, Venla Lehti, Minna Sucksdorff, Ona Sourander, Juha-Pekka Virtanen, Jutta Torsti, Andre Sourander

**Affiliations:** 1 Child and Youth Health Services, City of Helsinki, Helsinki, Finland; 2 National Institute for Health and Welfare, Helsinki, Finland; 3 Department of Child Psychiatry, Turku University Hospital, Turku, Finland; 4 Department of Pediatrics, Turku University Hospital, Turku, Finland; 5 Department of Psychiatry, University of Helsinki, Helsinki, Finland; 6 Helsinki University Hospital, Helsinki, Finland; 7 Research Center for Child Psychiatry, University of Turku, Lemminkäisenkatu 3/Teutori, 20014 Turku, Finland

**Keywords:** Learning disorders, Socioeconomic status, Maternal education, Marital status

## Abstract

**Background:**

Broadly defined learning and coordination disorders (LCDs) are common in the population and have previously been associated with familial social risk factors and male sex. However, comprehensive nationwide studies of these risk factors in LCD subgroups are lacking. Our objective was to assess different LCDs in relation to sex and maternal education, marital status and socioeconomic status based on occupation.

**Methods:**

We conducted a nationwide register-based study. The following diagnoses were identified from the Finnish Hospital Discharge Register (FHDR) according to the ICD-10 (*n* = 28,192): speech disorders (F80), scholastic disorders (F81), motor and coordination disorders (F82) and mixed developmental disorder (F83). To study cumulative incidence and male: female ratios of service use of LCDs, we used a cohort design among all Finnish children born singleton 1996–2007 (*n* = 690,654); to study social risk factors, we used a nested case-control design with extensive register data on both cases and matched controls (*n* = 106,616).

**Results:**

The cumulative incidence was 4.7% for any LCD by age 15 and the changes in cumulative incidence over time were minor. The male: female ratios were 2.2–3.0 across LCD subgroups. Learning and coordination disorders were more common in households with lower maternal education, socioeconomic status based on occupation and among children with single mothers at the time of birth; the odds ratios (OR) for any LCD were 1.2–1.9 across risk factors. The odds for LCD diagnosis increased linearly with the number of social risk factors, except for coordination disorder. The effect size of three risk factors was highest in the group with mixed or multiple LCDs; OR 3.76 (95% CI 3.31–4.28).

**Conclusions:**

Multiple social risk factors increase the odds for multiple, more comprehensive learning difficulties. The findings have implications for service planning, as early identification and interventions of learning and coordination disorders might reduce related long-term social adversities.

**Electronic supplementary material:**

The online version of this article (10.1186/s12889-018-5650-z) contains supplementary material, which is available to authorized users.

## Background

Learning and coordination disorders constitute a public health problem as they have prevalence estimates of 6–10% [1–3] and are associated with an increased risk for long-term problems related to education, work and mental health [4, 5]. To effectively identify and support the affected children and their families, health care and education resources need to be allocated correctly. Such service planning requires information on the proportion of subjects with a diagnosis, the age at which the diagnoses are given and the social risk factors [6]. Early interventions for developmental disorders have positive effects on cognitive, motor and long-term social development [7–9].

The definition and classification of learning disorders vary in the literature [1, 10–12]. For brevity, we use the umbrella term learning and coordination disorders (LCD) for developmental disorders of speech, scholastic skills and motor coordination. Children with LCDs have difficulties in specific cognitive processes but otherwise normal levels of intellectual functioning. The etiology of LCDs is multifactorial, with a strong but currently unspecified genetic component [13–15] in combination with pre- and postnatal environmental risk factors [1, 8, 16, 17].

Some studies have suggested learning and coordination disorders to be more common among children from families with social risk factors. In a survey-based study from the U.S [1], low parental education and poverty were associated with parent-reported learning disorders. Low maternal education and low household income has also been linked to impaired cognitive performance measured with the Differential Abilities Scale [8]. In a Finnish case-control study [16], low parental socioeconomic status (SES) increased the probability for children to receive special education in school. Among the children receiving special education because of borderline to mild intellectual disability, social risk factors were most common. Several studies have found LCDs to be approximately twice more common among boys than girls [1, 3, 18–20].

Longitudinal population-based data on social risk factors and the sex distribution of the whole spectrum of LCDs is not available to our best knowledge. We conducted a nationwide register-based study of all diagnosed LCDs in Finland among children born 1996 or later. Using a nested matched case-control design, our aim was to study the association between maternal education, marital status, SES based on occupation and diagnosed LCDs. Based on previous studies, we expected that LCDs are more common among children with single mothers and mothers with low education [1, 8, 16]. Further, to describe the temporal changes of diagnosed LCDs, the male: female ratios and the age of first diagnosis, we used a cohort design to report the cumulative incidence of specialized service use for LCDs. Compared to prevalence measures of service use, cumulative incidence measures have the advantage of defining at what age the cohort members are diagnosed for the first time. Previous survey-based studies of learning disabilities have reported higher prevalence among boys [1, 3] but no major changes in overall prevalence over time [3, 21], we therefore expected the cumulative incidence of diagnosed LCDs to be higher among boys and stable over time.

## Methods

### Participants and registers

The setting of the study is all singleton live births in Finland between 1996 and 2007 (*n* = 690,654). The study group utilized several Finnish nationwide registers and their information was linked via the personal identification code (PIC) of the subject. Ethical approval for the study was provided by the Ethics Committee of the Hospital District of Southwest Finland and the National Institute for Health and Welfare. The children were not contacted and therefore no informed consent was required according to Finnish law.

The number of live-born children was derived from the Finnish Medical Birth Register (FMBR) which contains information for all births in Finland since 1987. Data is available on maternal demographic characteristics and the data are virtually complete after data linkages to other governmental register resources [22].

Diagnostic information was derived from the Finnish Hospital Discharge Register (FHDR). This register contains information about inpatient care in all hospitals since 1969 and outpatient care in all public hospitals since 1998, including day of admission, main diagnosis and possible secondary diagnosis. Since 1996, all diagnoses are recorded according to the International Classification of Diseases (ICD-10) diagnostic classification. In previous studies, the overall diagnostic validity of the FHDR diagnoses has been good [23]. Information on social risk factors were obtained from the Statistics Finland Register and the FMBR. Statistics Finland is the Finnish public authority specifically established for statistics.

### Identification of learning and coordination disorders and matched controls

Finnish children undergo free routine health check-ups in public primary care by a trained nurse at least once a year and by a physician at least five times before entering school at the age of seven and three times later on, at ages 7, 11 and 14 [24, 25]. If a neurodevelopmental delay is suspected, children are referred to publicly funded specialist outpatient or inpatient clinics. The diagnosis of LCDs in Finland is usually based on multi-professional assessment in an outpatient clinic of pediatrics or pediatric neurology. Depending on the child’s difficulties, the diagnostic evaluation includes assessment by specialized nurse, pediatrician or pediatric neurologist, psychologist, speech therapist, occupational therapist and/or physiotherapist using standardized methods. For example, the psychological assessment includes Wechsler Preschool and Primary Scale of Intelligence or Wechsler Intelligence Scale for children. A more thorough neuropsychological assessment is done especially when diagnosing scholastic, speech and mixed LDs. Speech therapists use scales such as translations of Reynell Developmental Language Scales and Boston Naming Test for diagnosis. Physiotherapists often use the Movement Assessment Battery for Children.

In this study, we decided to include the whole spectrum of learning and coordination disorders grouped together in the ICD-10 classification as diagnostic codes F80-F83, i.e. those children diagnosed in specialized services with the following diagnoses: speech disorders (F80), scholastic disorders (F81), motor and coordination disorders (F82) and mixed developmental disorder (F83). Disorders of speech and language are conditions in which normal patterns of language acquisition are disturbed from the early stages of development. In scholastic disorders, reading, spelling or arithmetical skill acquisition development is impaired. Motor and coordination disorder refers to impaired fine and gross motor coordination development. Mixed developmental disorder is a category for disorders in which there is a mixture of specific developmental disorders of speech and language, scholastic skills, and motor function, but in which none predominates sufficiently to constitute the prime diagnosis. This mixed category is used when there is a major overlap between each of these specific developmental disorders and is usually, but not always, associated with a mild general impairment of cognitive functions [26].

**Fig. 1:**
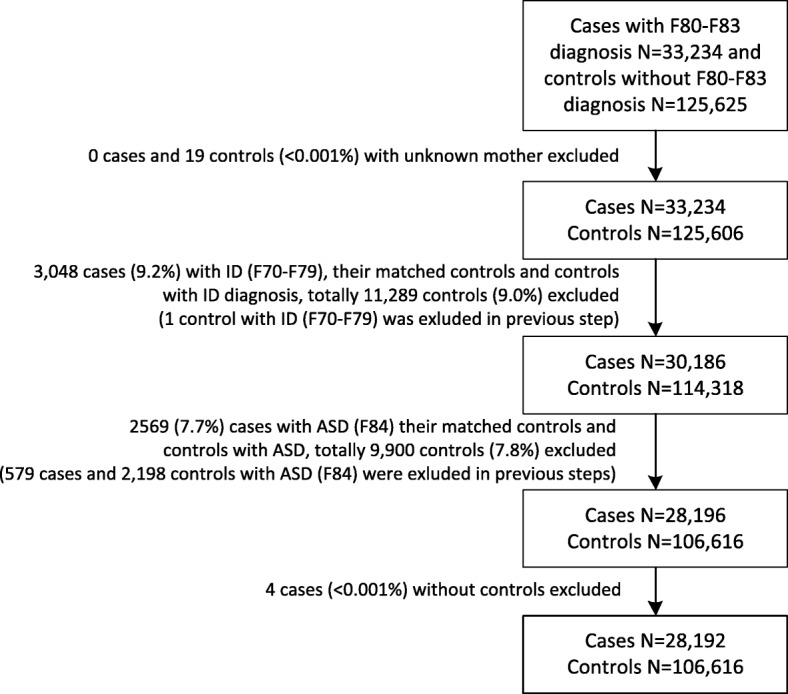
Flow chart of exclusion criteria in the nested case-control setting. ASD, autism spectrum disorder. ID, intellectual disability

Cases comprised children diagnosed with any or multiple LCDs (F80-F83) during follow-up (*n* = 33,234). As intellectual disability (ID) conflicts with the definition of learning disorder, 5038 children with co-occurring intellectual disability (F70–79) and autism spectrum disorders (ASD, F84) were excluded (*n* = 28,196). Of the excluded cases, the most common co-occurring learning disability was mixed developmental disorder (F83, *n* = 3159). Four cases that could not be matched with a control were excluded (n = 28,192). Each case was individually matched with four controls on sex and date of birth (+/− 30 days). Controls were defined as singleton born, alive and living in Finland at the time of matched case’s diagnosis, but without a diagnosis of LCD, ID or ASD (F80–83, F70–79, F84) (*n* = 106,616). For an overview of the exclusion criteria, see Fig. 1. In addition to studying any learning and coordination disorder, we studied the specific LCDs as mutually exclusive groups. We defined mutually exclusive groups in which cases could belong only to one group (see Table 1 for ICD-codes): speech disorder only, scholastic disorder only, coordination disorder only and mixed or ≥ 2 diagnostic classes.

**Table 1:** The diagnostic codes of learning and coordination disorders and the number of subjects diagnosed with any LCD and specific groups of LCDs by year 2012^a^

		Birth year (number born)		
		1996–1999 (*n* = 233,759)	2000–2003 (*n* = 224,105)	2004–2007 (*n*= 232,790)
Learning and coordination disorder	ICD-10 Code	No. (% of subjects with any LCD)	No. (% of subjects with any LCD)	No. (% of subjects with any LCD)
Any LCD, total	F80-F83	11,037	10,027	7132
Individual learning disorders ^b^				
Speech disorder	F80	5803 (52.6)	5954 (59.4)	4773 (66.9)
Scholastic skills disorder	F81	3665 (33.2)	2344 (23.4)	481 (6.7)
Coordination disorder	F82	2050 (18.6)	2193 (21.9)	1679 (23.5)
Mixed disorder	F83	2685 (24.3)	2611 (26.0)	2060 (28.9)
Mutually exclusive groups of LCDs				
Speech disorder only	F80, not F81-F83	3813 (34.5)	3936 (39.3)	3394 (47.6)
Scholastic disorder only	F81, not F80 or F82–83	2313 (21.0)	1318 (13.1)	237 (3.3)
Coordination disorder only	F82, not F80–81 or F83	910 (8.2)	941 (9.4)	749 (10.5)
Mixed	F83 and/or ≥ 2 diagnoses F80-F83	4001 (36.3)	3832 (38.2)	2752 (38.6)

Abbreviations: *LCD* learning and coordination disorder, *ICD-10* international classification of diseases, 10th edition

^a^Cases with co-occurring intellectual disability and autism spectrum disorders excluded

^b^The sum of subjects with individual learning disorders exceeds the number of subjects with any learning disorder because some subjects were diagnosed with multiple disorders

### Social risk factors

Data on maternal education was obtained from the Statistics Finland Register. Data on maternal SES based on occupation and marital status were derived from the FMBR. Education was classified as: 1) no college education (completed secondary school but no higher education) and 2) college education or higher (higher vocational or university degree). Mothers who have completed only comprehensive school are reported as missing in the register and subjects with missing information on education were assigned to the group with no college education. Marital status was divided into two groups: 1) married or in a relationship and 2) single, divorced or widowed. SES based on occupation was also divided into two groups following the Finnish national classifications on occupations and socio-economic groups [27, 28]: 1) white collar workers and higher 2) blue collar workers and others. Group 1 includes upper white-collar workers, for example people who work as upper clerical workers and leaders, experts or teachers. Group 1 also includes lower white-collar workers, which refers to lower clerical workers such as people doing office work, who are not leaders or experts. Group 2 includes blue-collar workers who perform manual labour and others, i.e. people outside the labour force, such as students, homemakers and unemployed people. Women who report education instead of occupation are classified as upper white-collar workers if they are known to have a university degree and as lower white-collar workers if they are known to have a lower than university level vocational degree. Education, marital status and SES were documented at the time of birth. We examined the frequency of the three studied maternal risk factors (‘no college education’, ‘single at the time of birth’ and ‘other SES than white collar worker’); this variable was classified as 0, 1, 2 and 3 maternal risk factors. We chose to study maternal variables as they have shown greater impact on offspring outcomes than paternal variables in previous studies [29].

### Cohort design for studying sex differences, age of first diagnosis and temporal changes

Because the nested case-control study was matched on sex and time of birth, the sex distribution, age of first diagnosis and temporal changes could not be studied using that design. Therefore, we used a cohort design to address these aims. In this design, we included information on sex and month of birth for all singleton births in Finland between 1996 and 2007 (*n* = 690,654). The age of the first diagnosis of learning or coordination disorders was used to study the cumulative incidence by sex for different LCDs (F80-F83), speech disorders (F80), scholastic disorders (F81), coordination disorders (F82) and mixed developmental disorder (F83).

### Statistical analyses

To study the cumulative incidence of learning and coordination disorders diagnosed in specialized services, we conducted time-to-event analyses in the cohort setting. The event was defined as the incidence of the studied diagnosis and separate analyses were conducted in which the event was any LCD, speech disorder, scholastic disorder, coordination disorder or mixed disorder. The entry time was at birth and subjects were censored at the time of the event or at the end of follow-up on December 31, 2012, whichever came first. To test for differences between the sexes and birth years, we conducted Cox regression analyses with sex and cohort (birth years 1996–1999, 2000–2003, 2004–2007) as the predictors, respectively. The male: female ratios were reported as exponential estimates derived from Cox regression analysis with 95% confidence intervals (CI).

To test for associations between social factors and LCDs in the matched case-control dataset, we conducted conditional logistic regression analyses. Separate analyses were conducted for case-control sets in which the case had any learning or coordination disorder and in which the case had speech disorder only, scholastic disorder only, coordination disorder only and mixed LCD or two or more LCDs. In univariate analyses, each social factor and the four-class categorical variable were entered separately in the model. In multivariate analyses, the three dichotomous maternal social factors were entered simultaneously in the model. The effect size of the associations between the social factors and the outcome were reported as odds ratios (OR) with 95% CIs. All analyses were conducted using R statistical software version 3.2.4.

## Results

Results for cumulative incidence, sex ratio and age of first diagnosis were obtained from the cohort design. Among 690,654 children born in Finland between 1996 and 2007, a total of 28,196 were diagnosed with LCDs in specialized health care by year 2012 (Table 1). In the 1996–1999 birth cohorts, i.e. the cohorts with the longest follow-up time, the cumulative incidence was 4.7% for any learning and coordination disorder by age 15 (Additional file 1: Table S1). Among children born 1996–1999, overlapping was common as 36.3% of the children with a learning or coordination disorder were diagnosed with a diagnosis of mixed LCD (F83) or two or more of any LCDs (F80–83) (Table 1). When examining children diagnosed with specific learning and coordination disorders without other LCD diagnoses in the 1996–1999 cohorts, 34.5% of the children were diagnosed with only speech disorder, 21.0% with only scholastic disorder and 8.2% with only coordination disorder (Table 1).

**Fig. 2:**
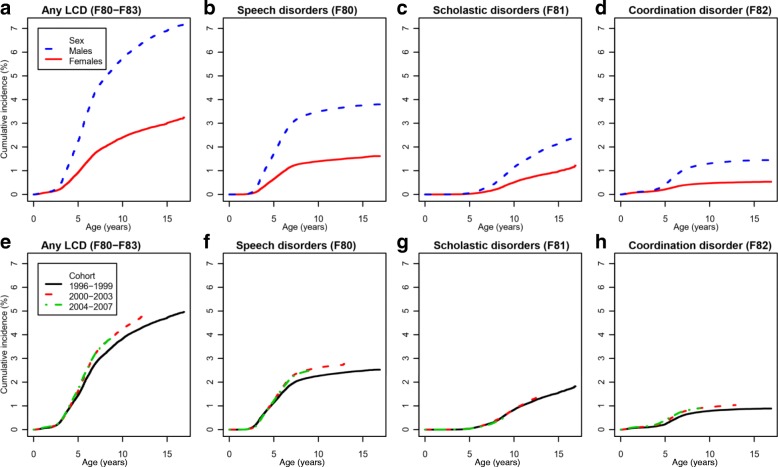
The cumulative incidence of learning and coordination disorders. The sex-stratified cumulative incidence of any LCD (panel **a**) and LCD subtypes (panels **b**-**d**) in specialized services. The birth-year stratified cumulative incidence of any LCD (panel **e**) and LCD subtypes (panels **f**-**h**) in specialized services. LCD, learning and coordination disorder

Figure 2 shows the cumulative incidence by sex (panels A-D) and birth year (panels E-H) in different types of LCDs. Boys were more likely to be diagnosed with any LCD than girls, and the sex ratio was similar across disorders with hazard ratios ranging between 2.20 and 2.95 (Additional file 1: Table S1). Changes in cumulative incidence over time were minor, e.g. the cumulative incidence for any LCD by age 10 increased from 3.8% in the 1996–1999 birth cohorts to 4.3% in the 2000–2003 birth cohorts (Fig. 2; Additional file 1: Table S1). The age of first diagnosis ranged between 5.3–9.8 years across disorders (Additional file 1: Table S1).

**Fig. 3:**
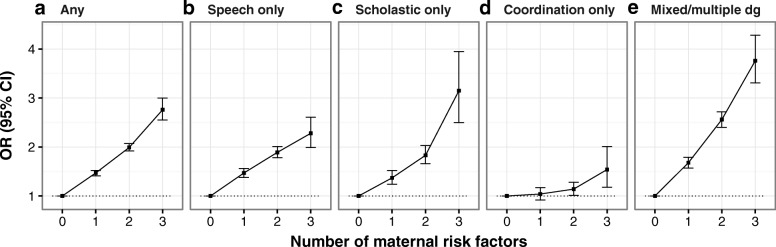
Odds ratios for the associations between social risk factors and learning and coordination disorder subgroups. Panel **a**) any learning and coordination disorder; **b**) speech disorder only; **c**) scholastic disorder only; **d**) coordination disorder only; and **e**) mixed or ≥ 2 diagnostic classes. Maternal risk factors: no college education, single at the time of birth, other SES than white collar. OR, odds ratio. CI, confidence interval

The social risk factors were studied in the matched case-control setting. Maternal education, marital status and SES based on occupation in relation to diagnosed learning and coordination disorders are shown in Table 2. LCDs were more common among children whose mother had low education, low SES or were single at the time of birth. The odds ratios from univariate and multivariate analyses for separate maternal risk factors in different learning and coordination disorders are shown in Table 3. All the diagnostic subgroups except coordination disorder showed independent associations with the risk factors in multivariate analyses, while coordination disorder was only associated with single motherhood (Table 3). When combining maternal risk factors, the odds of LCDs increased in a linear pattern (Fig. 3). The effect of multiple risk factors was strongest in the mixed LCD group: the OR of mixed LCDs was 3.76 (95% CI 3.31–4.28) for those with three risk factors compared to those with 0 risk factors. When the number of risk factors was examined as a continuous variable, the odds for any learning and coordination disorder diagnosis increased 41% by each number of risk factor (OR = 1.41, 95% CI 1.38–1.43).

**Table 2:** Maternal education, marital status and SES based on occupation in different learning and coordination disorders, birth cohorts 1996–2007

	Any learning or coordination disorder		Speech disorder only		Scholastic disorder only		Coordination disorder only		Mixed or ≥ 2 diagnostic classes	
	Cases	Controls	Cases	Controls	Cases	Controls	Cases	Controls	Cases	Controls
	(*n* = 28,192)	(*n* = 106,616)	(*n* = 11,142)	(*n* = 42,391)	(*n* = 3868)	(*n* = 14,945)	(*n* = 2600)	(*n* = 10,062)	(*n* = 10,582)	(*n* = 39,218)
Maternal characteristic	%	%	%	%	%	%	%	%	%	%
College education or higher										
Yes	29.0	42.9	31.1	43.9	27.7	40.7	38.2	41.9	24.9	42.9
No	71.0	57.1	68.9	56.1	72.3	59.3	61.8	58.1	75.1	57.1
Marital status at birth ^a^										
Married or in a relationship	92.1	95.0	93.0	94.7	92.1	95.8	92.2	95.2	91.0	94.8
Single or widowed	7.9	5.0	7.0	5.2	7.9	4.2	7.8	4.8	9.0	5.2
SES based on occupation at birth ^b^										
White collar workers or higher	52.2	62.6	53.3	62.6	53.7	62.5	60.7	62.2	48.3	62.7
Blue collar workers and others	47.8	37.4	46.7	37.4	46.3	37.5	39.3	37.8	51.7	37.3
Multiple risk factors ^c^										
None of the studied risk factors	24.9	36.9	26.2	37.6	25.4	35.6	33.6	36.2	21.2	36.9
1 of studied maternal risk factors	30.5	30.9	31.0	30.5	31.0	31.9	30.0	31.0	29.8	31.1
2 of studied maternal risk factors	40.1	29.7	39.0	29.5	39.2	30.5	32.6	30.2	43.4	29.5
3 of studied maternal risk factors	4.5	2.4	3.8	2.4	4.4	2.0	3.9	2.7	5.5	2.6

Abbreviations: *SES* socioeconomic status

^a^Missing data for maternal marital status: speech disorder 760 cases, 2771 controls, scholastic disorder 350 cases, 1266 controls, coordination disorder 175 cases, 697 controls, mixed disorder 804 cases, 2714 controls

^b^Missing data for maternal SES: speech disorder 1170 cases, 3909 controls, scholastic disorder 298 cases, 1139 controls, coordination disorder 203 cases, 898 controls, mixed disorder 1126 cases, 3679 controls

^c^Missing data for multiple risk factors: speech disorder 1758 cases, 6036 controls, scholastic disorder 590 cases, 2144 controls, coordination disorder 348 cases, 1432 controls, mixed disorder 1749 cases, 5766 controls

**Table 3:** Odds ratios for low maternal education, SES based on occupation and being single among children with different learning and coordination disorders, birth cohorts 1996–2007

		Maternal characteristic		
		No college education	Single at the time of birth	Other SES than white collar
Learning and coordination disorder		OR (95% CI) ^a^	OR (95% CI) ^a^	OR (95% CI) ^a^
Any LCD	Univariate	1.85 (1.80–1.90)	1.63 (1.54–1.72)	1.52 (1.48–1.56)
	Multivariate	1.62 (1.57–1.68)	1.37 (1.29–1.45)	1.24 (1.20–1.28)
Speech disorder	Univariate	1.74 (1.67–1.83)	1.36 (1.24–1.48)	1.46 (1.39–1.52)
	Multivariate	1.57 (1.49–1.66)	1.19 (1.08–1.31)	1.21 (1.15–1.28)
Scholastic disorder	Univariate	1.8 (1.67–1.95)	1.92 (1.65–2.24)	1.44 (1.33–1.55)
	Multivariate	1.61 (1.47–1.77)	1.62 (1.37–1.91)	1.15 (1.06–1.26)
Coordination disorder	Univariate	1.17 (1.07–1.28)	1.67 (1.39–2.00)	1.06 (0.96–1.16)
	Multivariate	1.11 (1.00–1.24)	1.56 (1.28–1.89)	0.98 (0.88–1.09)
Mixed disorder or > 2 diagnostic classes	Univariate	2.26 (2.16–2.38)	1.83 (1.68–1.99)	1.78 (1.70–1.87)
	Multivariate	1.89 (1.79–2.01)	1.44 (1.31–1.58)	1.37 (1.30–1.45)

Abbreviations: *LCD* learning and coordination disorder, *SES* socioeconomic status, *OR* odds ratio, *CI* confidence interval

^a^Calculated using conditional logistic regression analyses. All associations were significant at *p* ≤ 0.001 except for coordination disorder: other maternal SES than white collar univariate: *p* = 0.24, multivariate: *p* = 0.70 and no maternal college education multivariate *p* = 0.06. For number of subjects, see Table 2

## Discussion

The main findings of this study are that 1) the odds for all learning and coordination disorders except pure coordination disorder increase linearly with the number of social risk factors, 2) the effect of social risk factors is strongest in the group of mixed or multiple LCDs, suggesting clustering of social and learning problems, and 3) all learning and coordination disorders are more common among boys. Similar results have been observed in cohort studies when studying children’s psychiatric symptoms and their social risk factors [30, 31]. This study adds to the literature by examining social risk factors in specific learning and coordination disorders.

The reasons for the socioeconomically unequal distribution of LCDs and overall poorer academic achievement among children have been proposed to be both genetic and environmental [1, 8, 17]. One likely explanation is that parents with learning and coordination disorders are more likely to have low education [4], and due to genetic predisposition and differences in the home environment, also more likely to have children with LCDs. These children in turn are at greater risk for social exclusion and low SES in adulthood [4, 5], creating a vicious cycle. Pre- and postnatal environmental risk factors such as smoking and substance abuse may also partly explain the finding, as these factors are associated with LCDs [32–34] and are more common among mothers with low SES [35]. In the 1990s, the term “word gap” was introduced by Hart et al. [36]. They found a difference of approximately 30 million words addressed to three-year-old children from high SES families versus low SES families. Similar results have been replicated in larger samples and in children as young as 18 months [37]. In a prospective study from the U.S [8], higher SES, reading aloud to the child and having puzzles in the home were associated with higher scores in the Differential Ability Scales among pre-school children.

The association between social risk factors and LCDs was most pronounced among children with more pervasive difficulties, i.e. the mixed LCD and ≥ 2 diagnostic classes group. This finding is consistent with a previous Finnish case-control study [16], implicating that children from less advantaged backgrounds presenting with developmental difficulties need to be thoroughly evaluated, as risks for adversities tend to cluster [38]. A practical example of implementing this could be to consider regional differences in parental education and SES, when planning and providing resources for educational and health care services.

As expected based on previous studies, we found LCD diagnoses to be 2–3 times more common among boys than among girls. Some studies implicate that the genetic vulnerability is higher among males compared to females [39]. Others have suggested that health and education professionals have lower thresholds to refer boys than girls to special education or specialized health care [40]. We did not have information on referral practices, but the systematized check-ups in Finland and the consistent sex-ratio suggests that referral bias is unlikely to explain the sex-difference completely.

The group with pure coordination disorder was not as independently associated with low parental education or low maternal SES as the other types of LCD were. Although previous research on the subject is scarce, a smaller cohort study from the U.K. [41] found low maternal socioeconomic status to be associated with a 1.6-fold risk of developmental coordination disorder. However, only known neurologic conditions and children with an IQ < 70 were excluded from the study, suggesting possible overlap with other LCD diagnoses to explain the finding. In this study, those with coordination disorder and some other learning disorder belonged to the mixed group.

The age of first diagnosis was found to be somewhat delayed compared to the typical appearance of learning difficulties, a finding coherent with previous studies [1]. This is a problem when considering the need and benefits of early interventions. Though children might receive extra support also without a diagnosis, the diagnosis usually ensures it. In line with previous studies [3], we also found only a small increase in the cumulative incidence of learning and coordination disorders diagnosed in specialized healthcare by year 2012 in Finland.

The strengths of this study include the longitudinal nationwide sample of diagnosed LCDs and a uniform diagnostic system (ICD-10). To our knowledge, no nationwide studies have previously been conducted worldwide regarding the whole spectrum of learning and coordination disorders and their social risk factors. Due to the large sample size, we could separately study specific learning and coordination disorders in mutually exclusive groups and mixed or multiple learning disorders in relation to the accumulation of social risk factors. The limitations of this study are: 1) the study included only diagnoses from specialized services, which may result in missing of cases that never reach specialized services. However, it is unlikely that the relation of cases with higher and lower socioeconomic status, single motherhood and education status is affected by this, because of the systematic screening of all Finnish children via health check-ups that are free of charge. The cumulative incidence of LCDs in this study was also similar to studies with parent-reported prevalence numbers [3, 21]. 2) We did not have information on the validity of the register-based LCD diagnoses. However, due to the use of standardized tools when conducting diagnoses in specialized services, we estimated the risk of invalid diagnoses to be low. Diagnoses derived from the FHDR register have previously shown good validity in e.g. ADHD, autism and Tourette’s syndrome [42–44]. 3) The register-based sample limited the number of social variables possible to study. Information on e.g. household income, detailed family structure and paternal variables would have enabled a more comprehensive risk analysis. Furthermore, we wanted to maximize interpretability and therefore did not include sociobiological variables such as maternal age and parity. Further studies are needed to unravel the complex effects of social, biological and environmental risk factors on LCDs.

## Conclusions

Learning disorders except coordination disorder are more common in less advantaged households, especially households with multiple social risk factors, and among boys. Social risk factors are important to consider when studying other potential risk factors for LCDs. These findings have implications for the planning of healthcare and education services. Special attention needs to be addressed to families with single mothers, low education and low socioeconomic status. Early identification and a smooth referral system are important to ensure the help and support needed for the children and their families. Timely interventions of learning and coordination disorders can potentially reduce the related long-term social adversities such as social exclusion and unfinished education that cause individual suffering and a financial burden for society.

## Additional file

Additional file 1: **Table S1.** Cumulative incidence, median age of first diagnosis and sex ratio of learning and coordination disorders, birth cohorts 1996–2007. (DOCX 20 kb)

## Abbreviations

ADHD: Attention-deficit/hyperactivity disorder
ASD: Autism spectrum disorder
CI: Confidence interval
FHDR: Finnish Hospital Discharge Register
FMBR: Finnish Medical Birth Register
HR: Hazard ratio
ICD: International Classification of Diseases
ID: Intellectual disability
IQ: Intelligence quotient
LCD: Learning and coordination disorder
OR: Odds ratio
PIC: Personal identification code
SES: Socioeconomic status
