# Supplementary material for: Social risk factors for speech, scholastic and coordination disorders: a nationwide register-based study
Source: BMC Public Health. 2018 Jun 15;18:739. doi: 10.1186/s12889-018-5650-z (PMC6002992; doi:10.1186/s12889-018-5650-z)
Supplement: Supplementary file 1 — Table S1. Cumulative incidence, median age of first diagnosis and sex ratio of learning and coordination disorders, birth cohorts 1996–2007. (DOCX 20 kb) [file 12889_2018_5650_MOESM1_ESM.docx]

**Table S1 Cumulative incidence, median age of first diagnosis and sex ratio of learning and coordination disorders, birth cohorts 1996-2007.^a, b^**

|  |  | **Age of first diagnosis** | **Cumulative incidence by age 5** | **Cumulative incidence by age 10** | **Cumulative incidence by age 15** | **Male: Female ratio** |
| --- | --- | --- | --- | --- | --- | --- |
| **Learning and coordination disorder** | Birth year | Median (IQR) | 1/100 (95% CI) | 1/100 (95% CI) | 1/100 (95% CI) | HR (95% CI) ^c^ |
| **Any LCD** | 1996-1999 | 6.25 (4.50-9.00) | 1.46 (1.41-1.51) | 3.83 (3.75-3.91) | 4.71 (4.63-4.80) | 2.42 (2.32-2.52) ^d^ |
|  | 2000-2003 | 5.67 (4.25-7.25) | 1.64 (1.59-1.70) | 4.26 (4.17-4.34) |  |  |
|  | 2004-2007 | 4.75 (3.67-5.75) | 1.71 (1.66-1.77) |  |  |  |
| **Speech disorder** | 1996-1999 | 5.25 (3.75-6.75) | 1.15 (1.11-1.19) | 2.26 (2.20-2.32) | 2.48 (2.42-2.55) | 2.50 (2.36-2.65) ^d^ |
|  | 2000-2003 | 5.17 (3.94-6.41) | 1.24 (1.19-1.28) | 2.61 (2.55-2.68) |  |  |
|  | 2004-2007 | 4.59 (3.67-5.66) | 1.23 (1.18-1.27)  0.04 (0.03-0.05) |  |  |  |
| **Scholastic disorder** | 1996-1999 | 9.75 (8.17-12.00) |  | 0.84 (0.80-0.88) | 1.55 (1.50-1.61) | 2.20 (2.05-2.37) ^d^ |
|  | 2000-2003 | 8.59 (7.25-9.75) | 0.05 (0.04-0.06) | 0.87 (0.83-0.91) |  |  |
|  | 2004-2007 | 6.42 (5.41-7.42) | 0.04 (0.03-0.05) |  |  |  |
| **Coordination disorder** | 1996-1999 | 6.08 (5.00-7.56) | 0.23 (0.21-0.25) | 0.79 (0.75-0.82) | 0.88 (0.84-0.92) | 2.95 (2.67-3.26) ^d^ |
|  | 2000-2003 | 5.67 (4.42-6.83) | 0.32 (0.30-0.35) | 0.95 (0.91-0.99) |  |  |
|  | 2004-2007 | 4.84 (3.66-5.75) | 0.39 (0.36-0.41) |  |  |  |
| **Mixed disorder** | 1996-1999 | 6.41 (5.25-8.50) | 0.25 (0.23-0.27) | 0.95 (0.91-0.99) | 1.15 (1.10-1.19) | 2.49 (2.29-2.71) ^d^ |
|  | 2000-2003 | 5.92 (4.83-7.25) | 0.32 (0.30-0.35) | 1.11 (1.07-1.15) |  |  |
|  | 2004-2007 | 5.17 (4.25-6.00) | 0.39 (0.36-0.41) |  |  |  |
| Abbreviations: CI, confidence interval; HR, hazard ratio, IQR, inter-quartile range, LCD, learning and coordination disorder.  ^a^ Analyses were conducted using time-to-event analysis. The ICD-10 codes for the individual disorders are shown in Table 1.  ^b^ Cases with co-occurring intellectual disability and autism spectrum disorders were excluded.  ^c^ Calculated using Cox regression analysis. | | | | | | |
| ^d^ p-value <0.001 |  |  |  |  |  |  |
